# Geraniol alleviates LPS-induced acute lung injury in mice via inhibiting inflammation and apoptosis

**DOI:** 10.18632/oncotarget.20298

**Published:** 2017-08-16

**Authors:** Kangfeng Jiang, Tao Zhang, Nannan Yin, Xiaofei Ma, Gan Zhao, Haichong Wu, Changwei Qiu, Ganzhen Deng

**Affiliations:** ^1^ Department of Clinical Veterinary Medicine, College of Veterinary Medicine, Huazhong Agricultural University, Wuhan 430070, People’s Republic of China

**Keywords:** acute lung injury, geraniol, inflammation, apoptosis, NF-κB

## Abstract

Geraniol (GOH), a special type of acyclic monoterpene alcohol, has been widely used to treat many diseases associated with inflammation and apoptosis. Acute lung injury (ALI) is a common clinical disease in humans characterized by pulmonary inflammation and apoptosis. In the present study, we investigated the protective effects of GOH in a mouse model of ALI induced by the intranasal administration of lipopolysaccharide (LPS) and elucidated the underlying molecular mechanisms in RAW 264.7 cells. *In vivo*, GOH treatment markedly ameliorated pathological injury and pulmonary cell apoptosis and reduced the wet/dry (W/D) weight ratio of lungs, myeloperoxidase (MPO) activity and the production of pro-inflammatory cytokines (IL-1β, IL-6, and TNF-α). *In vitro*, the levels of pro-inflammatory cytokines, iNOS and COX-2 were significantly increased in LPS-stimulated RAW 264.7 cells, an effect that was decreased by GOH treatment. Moreover, GOH treatment dramatically reduced the expression of Toll-like receptor 4 (TLR4) and then prevented the nuclear factor-κB (NF-κB) activation. GOH treatment also promoted anti-apoptotic Bcl-2 expression and inhibited pro-apoptotic Bax and Caspase-3 expression. Furthermore, knockdown of TLR4 expression exerted a similar effect and inhibited the phosphorylation of p65, as well as the Bax and Caspase-3 expression. Taken together, these results suggest that GOH treatment alleviates LPS-induced ALI via inhibiting pulmonary inflammation and apoptosis, a finding that might be associated with the inhibition of TLR4-mediated NF-κB and Bcl-2/Bax signalling pathways.

## INTRODUCTION

Acute respiratory distress syndrome (ARDS), one of the severe complications of acute lung injury (ALI), remains a major cause of morbidity and mortality in critically ill patients with ALI [1–3]. ALI and ARDS are characterized by an acute pulmonary inflammatory response and are associated with various clinical disorders, such as pneumonia, interstitial oedema and sepsis [4]. The pathogenesis of ALI/ARDS mainly involves exaggerated pulmonary inflammation, ultimately leading to an impairment of the alveolar-capillary barrier and deterioration of gas exchange [5, 6]. Moreover, apoptosis is another critical factor of ALI and plays a vital role in the development of ALI [7, 8].

Various inflammatory stimuli produced by pathogenic microorganisms have been generally recognized for their ability to cause pulmonary inflammation [9, 10]. Lipopolysaccharide (LPS), a major biologically active component of the Gram-negative bacterial cell wall, plays a crucial role in lung inflammation, and the intranasal administration of LPS has long been widely used to induce pulmonary inflammation in a mouse model of ALI [11–13]. Additionally, LPS can also activate pro-apoptotic signals in many different types of cells, including macrophages, epithelial cells and endothelial cells [14–16]. Toll-like receptor 4 (TLR4), a main receptor of LPS, is involved in the initiation and acceleration of inflammatory responses induced by LPS and can induce pro-apoptotic pathways that lead to cell death [16, 17].

**Figure 1:**
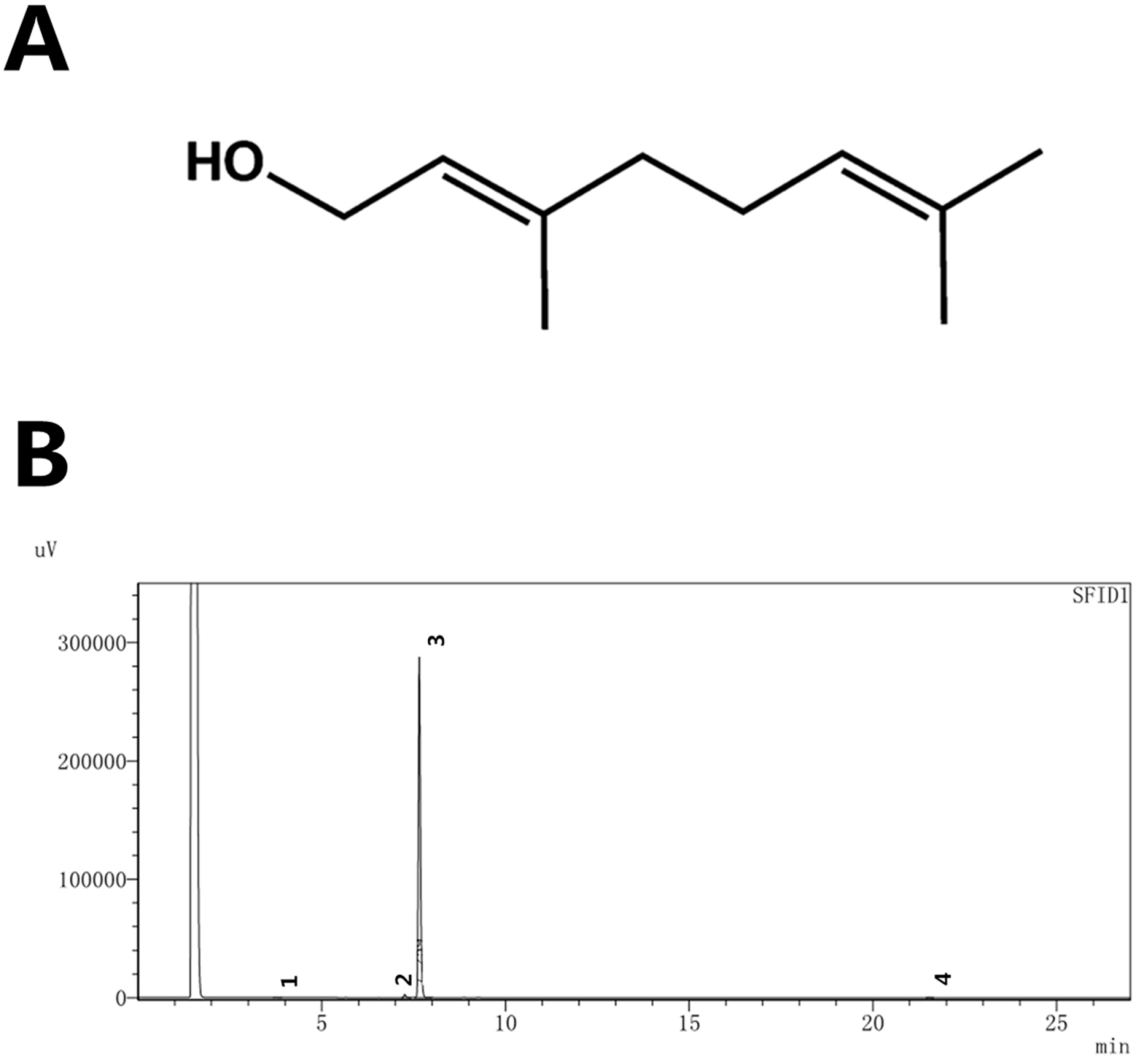
**(A)** Chemical structure of GOH, **(B)** HPLC chromatogram of GOH.

Geraniol (GOH; Figure 1A), a type of acyclic monoterpene alcohol, is a monomer mainly extracted from the essential oils of lemon, rose, ginger, orange, among others [18]. Some research results concerning the pharmacological activities of GOH have shown that it possesses anti-inflammatory [19], anti-apoptotic [20] and anti-tumoural properties [21]. Whether GOH has anti-inflammatory and anti-apoptotic effects in LPS-induced ALI, however, remains unclear. Therefore, the present study was designed to investigate whether GOH attenuates ALI induced by LPS through the down-regulation of pulmonary inflammation and apoptosis and illustrate the underlying mechanisms.

## RESULTS

### *In vivo* study

#### GOH treatment attenuates LPS-induced lung injury in mice

**Figure 2:**
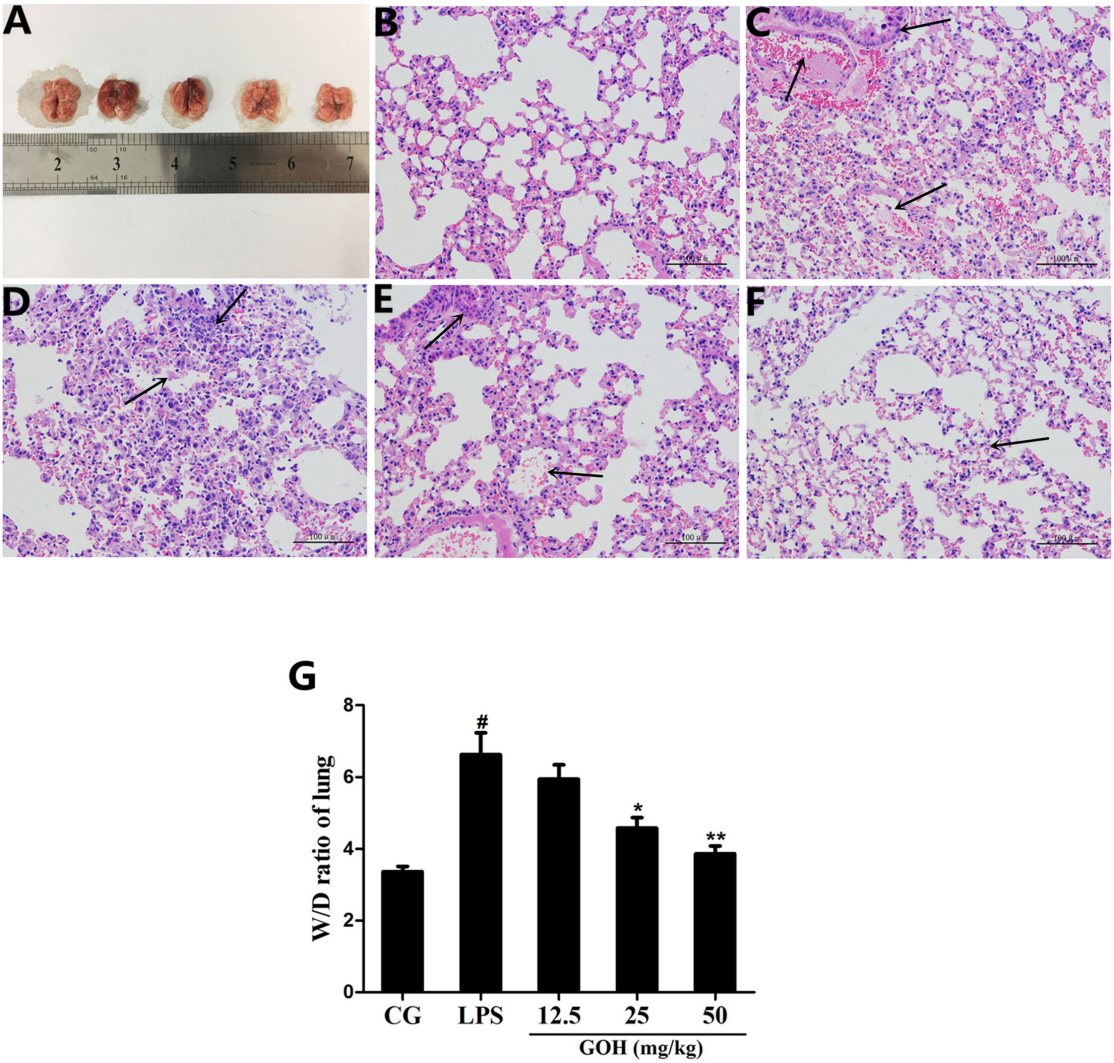
Effects of GOH on LPS-induced lung injury **(A)** Morphology of the lung. **(B)** Control group, **(C)** LPS group, **(D-F)** GOH (12.5, 25, and 50 mg/kg) groups. The black arrows indicate the tissue lesion area. **(G)** Lung W/D ratio. CG is the control group. LPS is the LPS-stimulated group. Data represent means ± S.E.M. of three independent experiments. #p< 0.05 vs. the control group. *p < 0.05 vs. the LPS group, **p<0.01 vs. LPS group.

The morphology of the lungs from each group was observed (Figure 2A). Next, the effects of GOH on lung histopathology were determined using H&E staining. As shown in Figure 2, the lungs of mice exposed to LPS showed significant lung injury characterized by pulmonary oedema, inflammatory cell infiltration and alveolar damage (Figure 2C). However, GOH treatment significantly improved lung injury (Figure 2D-2F). There were no obvious pathological changes in the control group (Figure 2B). In addition, LPS-challenged mice have a dramatic increase in the lung wet/dry (W/D) weight ratio relative to the control group that was reduced by GOH treatment (Figure 2G).

#### GOH treatment decreases LPS-induced myeloperoxidase (MPO) activity

**Figure 3:**
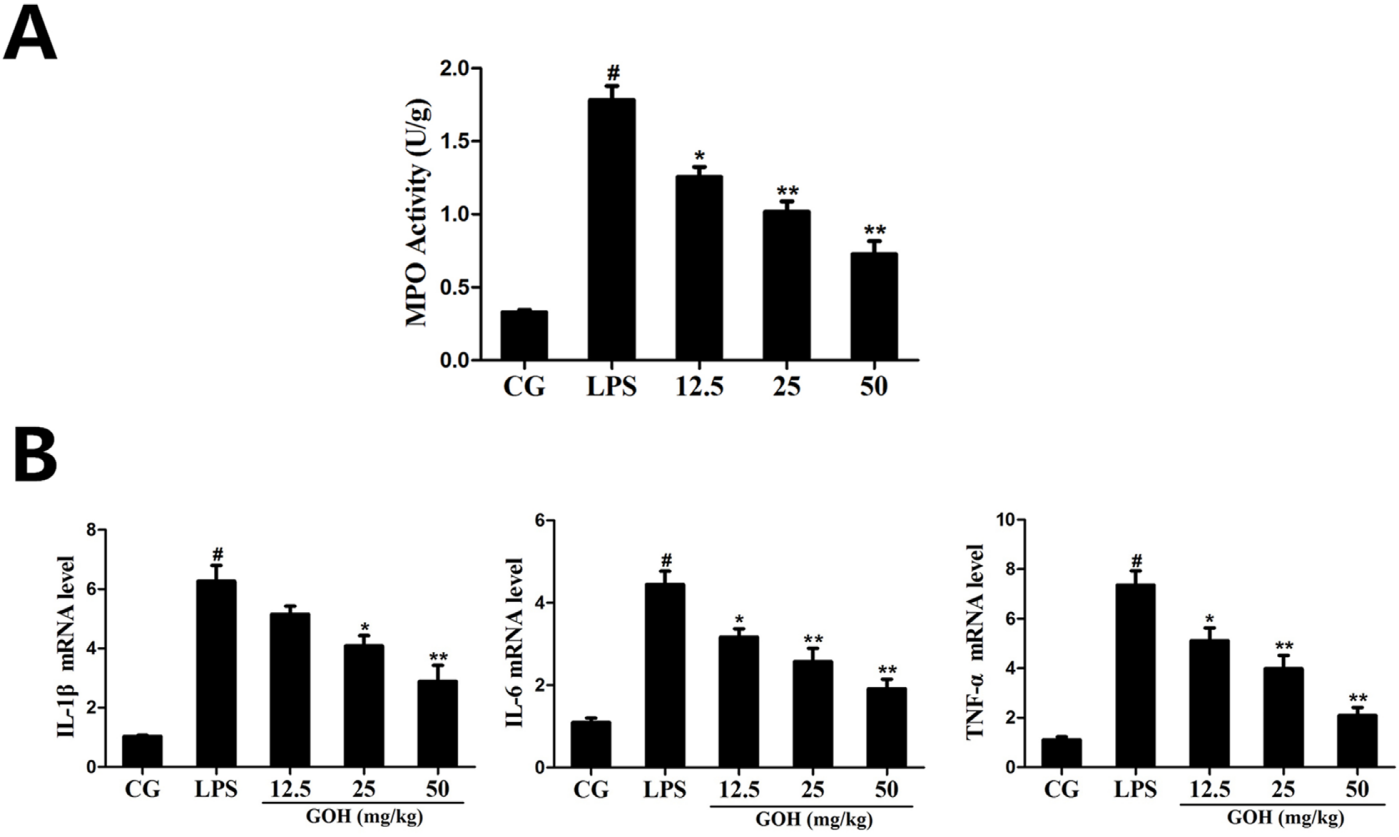
Effects of GOH on MPO activity and cytokine production in lung tissues **(A)** MPO activity assay. **(B)** The expression of TNF-α, IL-1β, and IL-6 mRNA in tissues was measured by qPCR. GAPDH was used as a control. CG is the control group. LPS is the LPS-stimulated group. The values are presented as means ± S.E.M. of three independent experiments. #p< 0.05 vs. the control group. *p< 0.05 vs. the LPS group, **p<0.01 vs. LPS group.

To assess the neutrophil infiltration in the lung tissues, we measured the lung MPO activity. As Figure 3A shows, LPS significantly increased the MPO activity in the lung tissues compared with that in the control group. GOH treatment obviously inhibited the increased MPO activity induced by LPS.

#### GOH treatment downregulates the production of inflammatory cytokines in tissues

The expression of the inflammatory cytokines (IL-1β, IL-6, and TNF-α) in tissues was determined using the qPCR assay. As shown in Figure 3B, we found that the expression of IL-1β, IL-6, and TNF-α was significantly increased in the LPS group. By contrast, GOH treatment dose dependently downregulated the expression of these cytokines. These results indicated that GOH treatment may inhibit pulmonary inflammation in mice.

#### GOH treatment ameliorates the apoptosis in LPS-induced ALI

**Figure 4:**
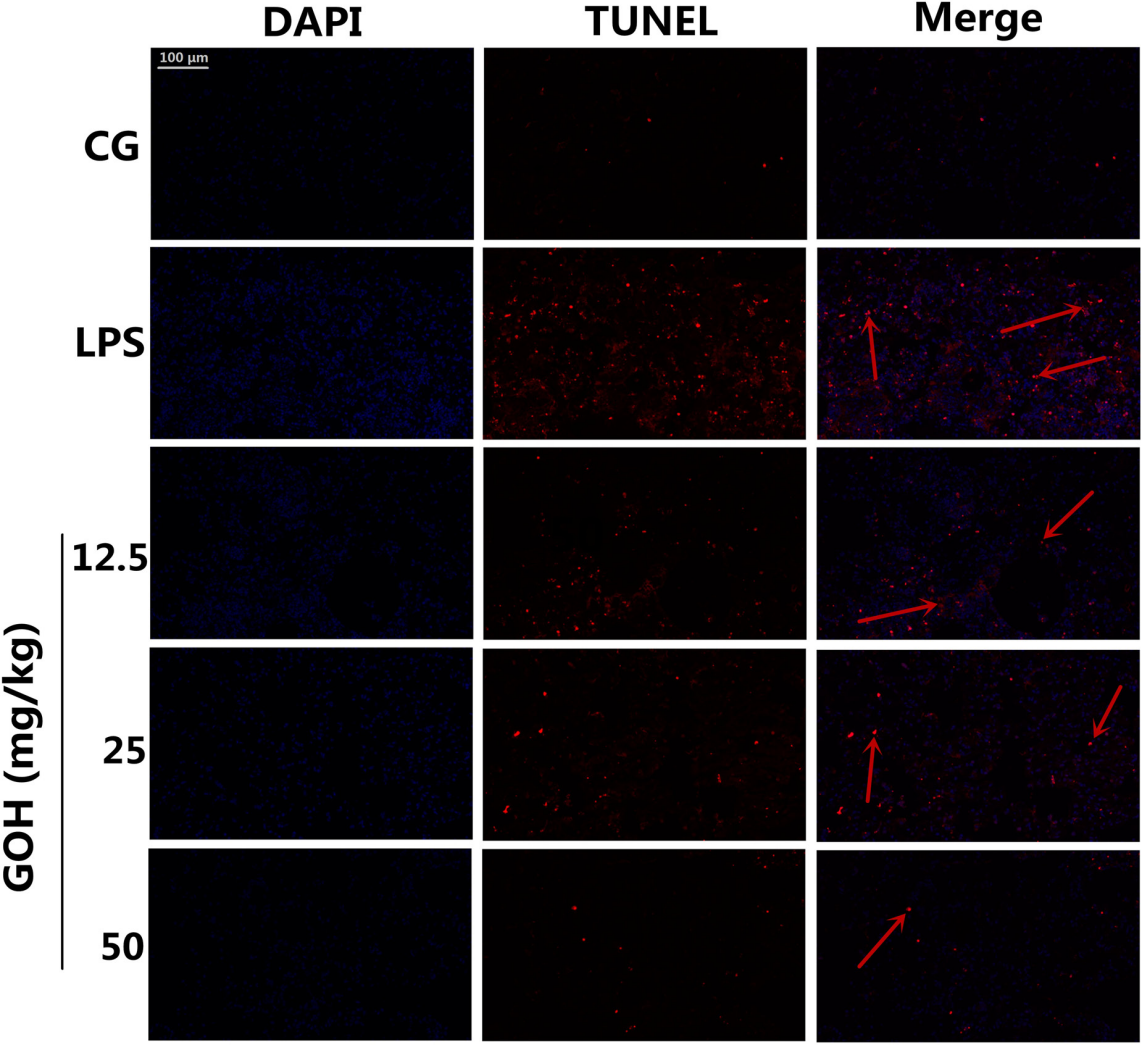
Apoptosis detection of LPS-induced lung injury 24 h after LPS infection, apoptotic cells in lung tissues were detected using dual TUNEL and DAPI staining. Scale bar: 100 μm. The red arrows indicate the apoptotic region. Blue cells were nonapoptotic cells, and those with red nuclei were apoptotic cells.

In the study, we also investigated the anti-apoptotic effect of GOH in LPS-induced ALI by the TUNEL assay. Numerous apoptotic cells appeared in the lung tissues of LPS-challenged mice. In the GOH treatment groups, however, a few of the lung cells were positive for TUNEL staining (Figure 4). These results indicated that GOH treatment may alleviate lung cell apoptosis in mice.

### *In vitro* study

#### Effect of GOH treatment on cell viability

**Figure 5:**
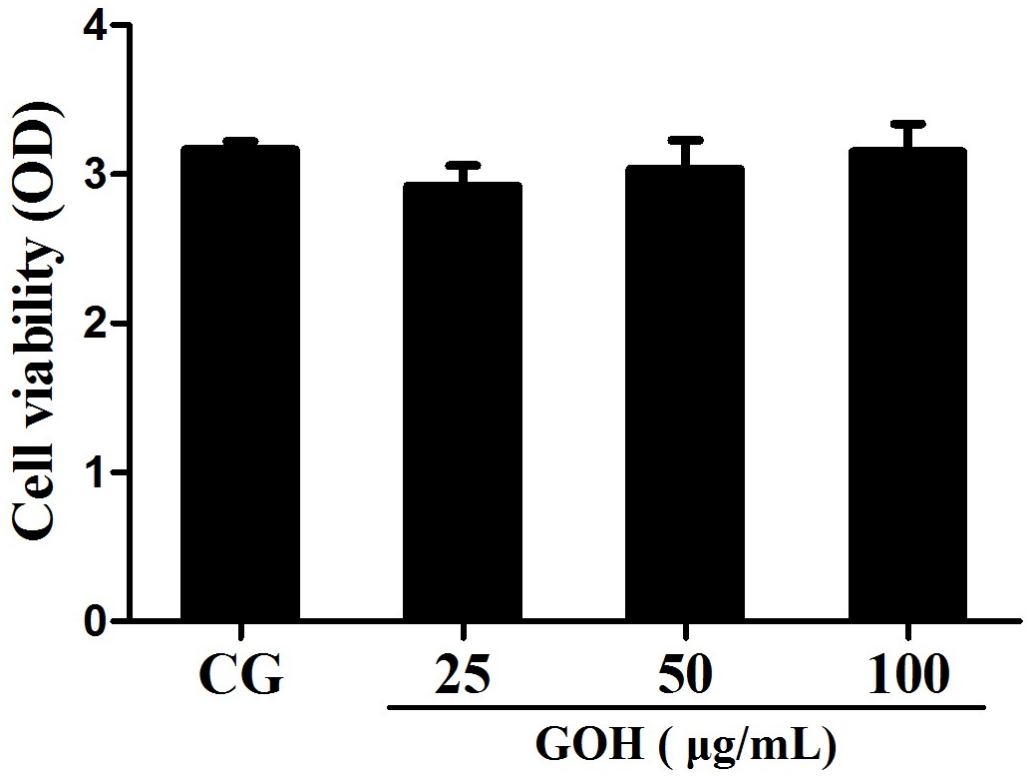
Effects of GOH on the cell viability RAW 264.7 cells were cultured with different concentrations of GOH (25, 50, and 100 μg/mL) for 24 h, and then the cell viability was measured using the MTT assay. The values are presented as means ± S.E.M. of three independent experiments. #p< 0.05 vs. the control group. *p<0.05 vs. the LPS group, **p<0.01 vs. LPS group.

To investigate whether GOH was cytotoxic to RAW 264.7 cells, we first examined its effects on cell viability by the MTT assay. These results demonstrated that the cell viability was not affected by GOH treatment (Figure 5).

#### GOH treatment downregulates the production of inflammatory cytokines in RAW 264.7 cells

**Figure 6:**
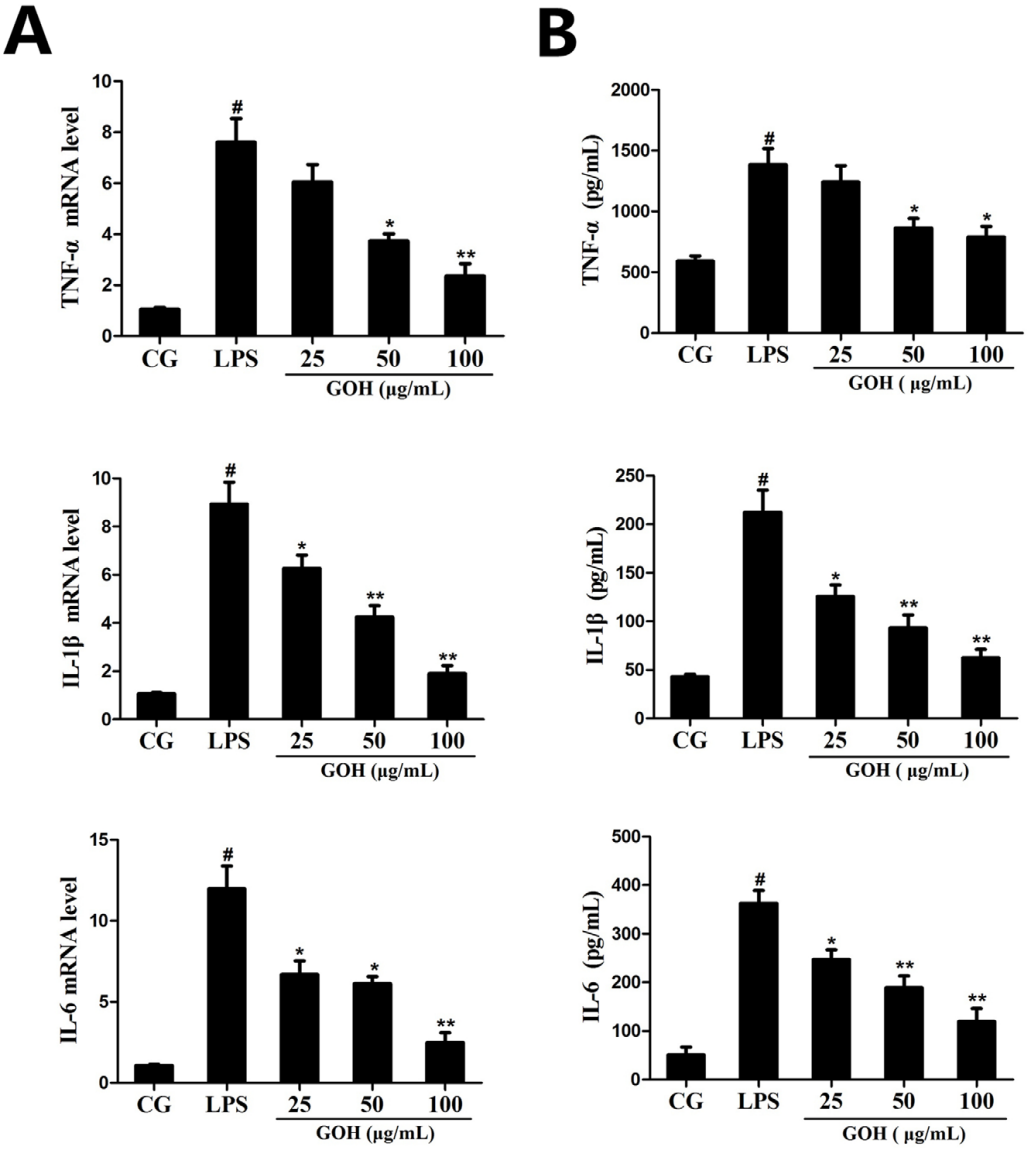
Effects of GOH on the production of cytokines in LPS-stimulated RAW 264.7 cells **(A)** The expression of TNF-α, IL-1β, IL-6 mRNA was detected by qPCR. GAPDH was used as a control. **(B)** The levels of TNF-α, IL-1β, and IL-6 proteins were measured by ELISA. CG is the control group. LPS is the LPS-stimulated group. The values are presented as means ± S.E.M. of three independent experiments. #p<0.05 vs. the control group. *p< 0.05 vs. the LPS group, **p<0.01 vs. the LPS group.

*In vivo* experiments implicated that GOH may have a potential anti-inflammatory effect. To confirm the results, the levels of TNF-α, IL-1β, and IL-6 in cells were determined by qPCR and ELISA. These results showed that the expression of TNF-α, IL-1β and IL-6 was dramatically increased after LPS stimulation. By contrast, GOH treatment dose dependently reduced these increases (Figure 6A and 6B).

#### GOH treatment reduces the production of iNOS and COX-2 in RAW 264.7 cells

**Figure 7:**
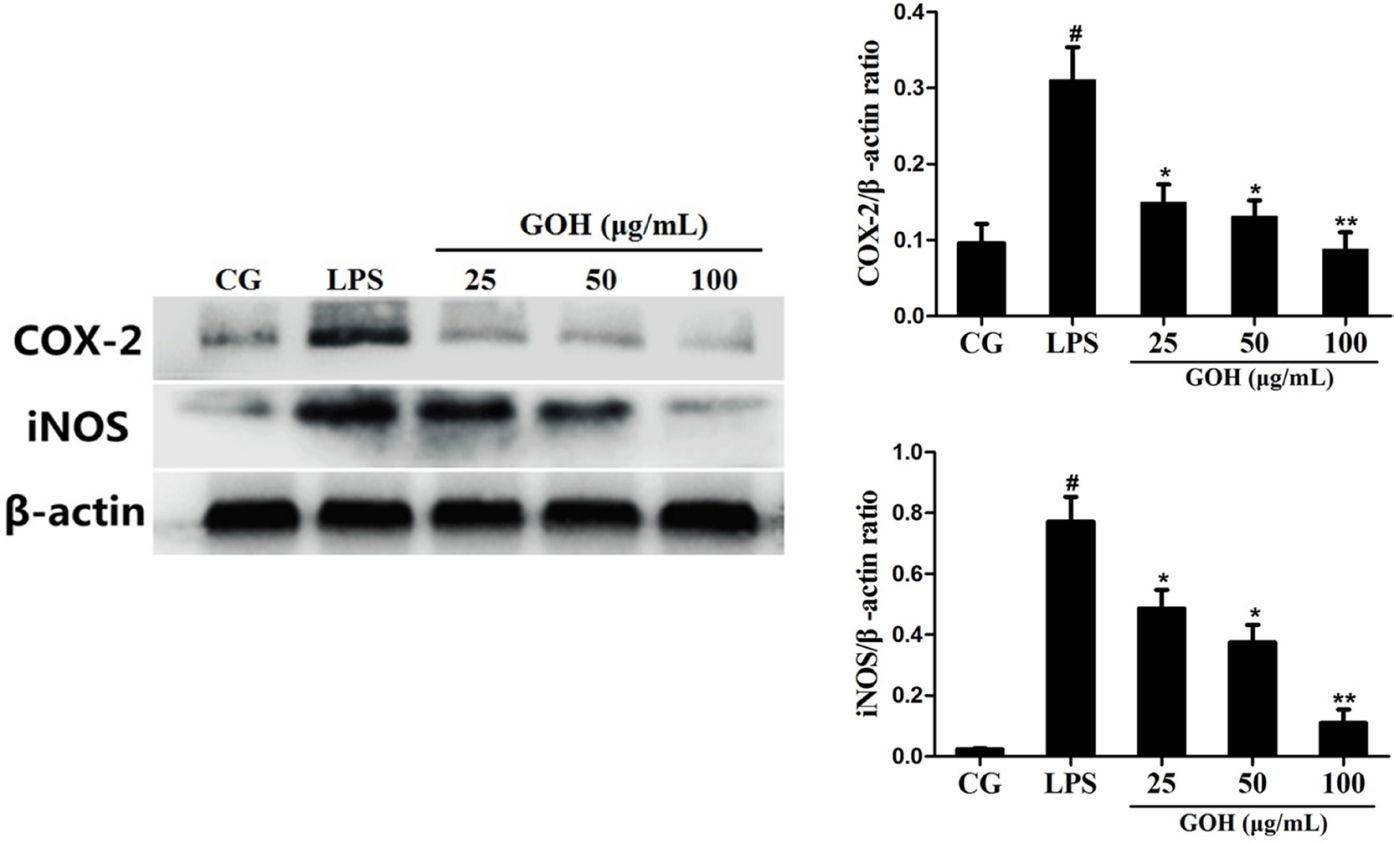
Effects of GOH on the levels of iNOS and COX-2 proteins in LPS-stimulated RAW 264.7 cells The levels of iNOS and COX-2 proteins were measured by western blotting. CG is the control group. LPS is the LPS-stimulated group. The values are presented as means ± S.E.M. of three independent experiments. #p< 0.05 vs. the control group. *p< 0.05 vs. the LPS group, **p<0.01 vs. the LPS group.

The inflammatory mediators iNOS and COX-2 reflect the state of inflammation and are often used to evaluate the severity of inflammation [22]. As shown in Figure 7, stimulation with LPS led to a significant increase in iNOS and COX-2 expression. However, the expression of iNOS and COX-2 was downregulated by GOH treatment. These results suggest that GOH plays an anti-inflammatory role in LPS-stimulated RAW 264.7 cells.

#### GOH treatment decreases the expression of TLR4 in RAW 264.7 cells

**Figure 8:**
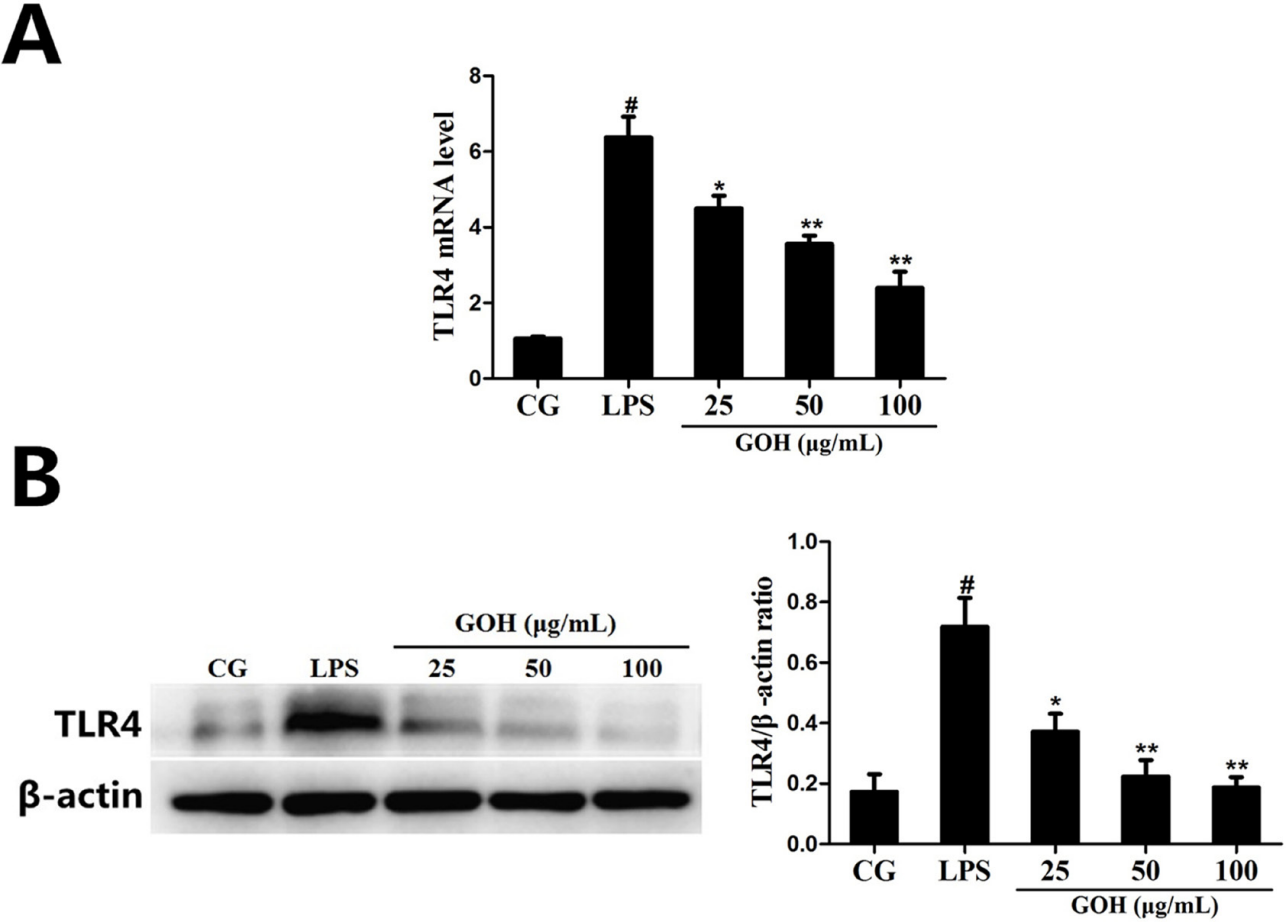
The effects of GOH on the expression of TLR4 in LPS-stimulated RAW 264.7 cells **(A)** The expression of TLR4 mRNA was determined by qPCR. GAPDH was used as a control. **(B)** The level of TLR4 protein was measured by western blot. CG is the control group. LPS is the LPS-stimulated group. The values are presented as means ± S.E.M. of three independent experiments. #p<0.05 vs. the control group. *p<0.05 vs. the LPS group, **p<0.01 vs. the LPS group.

To detect whether GOH could affect TLR4, the expression of TLR4 in LPS-stimulated RAW 264.7 cells was measured using qPCR and western blotting. As shown in Figure 8A and 8B, the expression of TLR4 induced by LPS was significantly inhibited by GOH treatment.

#### GOH treatment inhibits LPS-induced activation of the NF-κB pathway in RAW 264.7 cells

**Figure 9:**
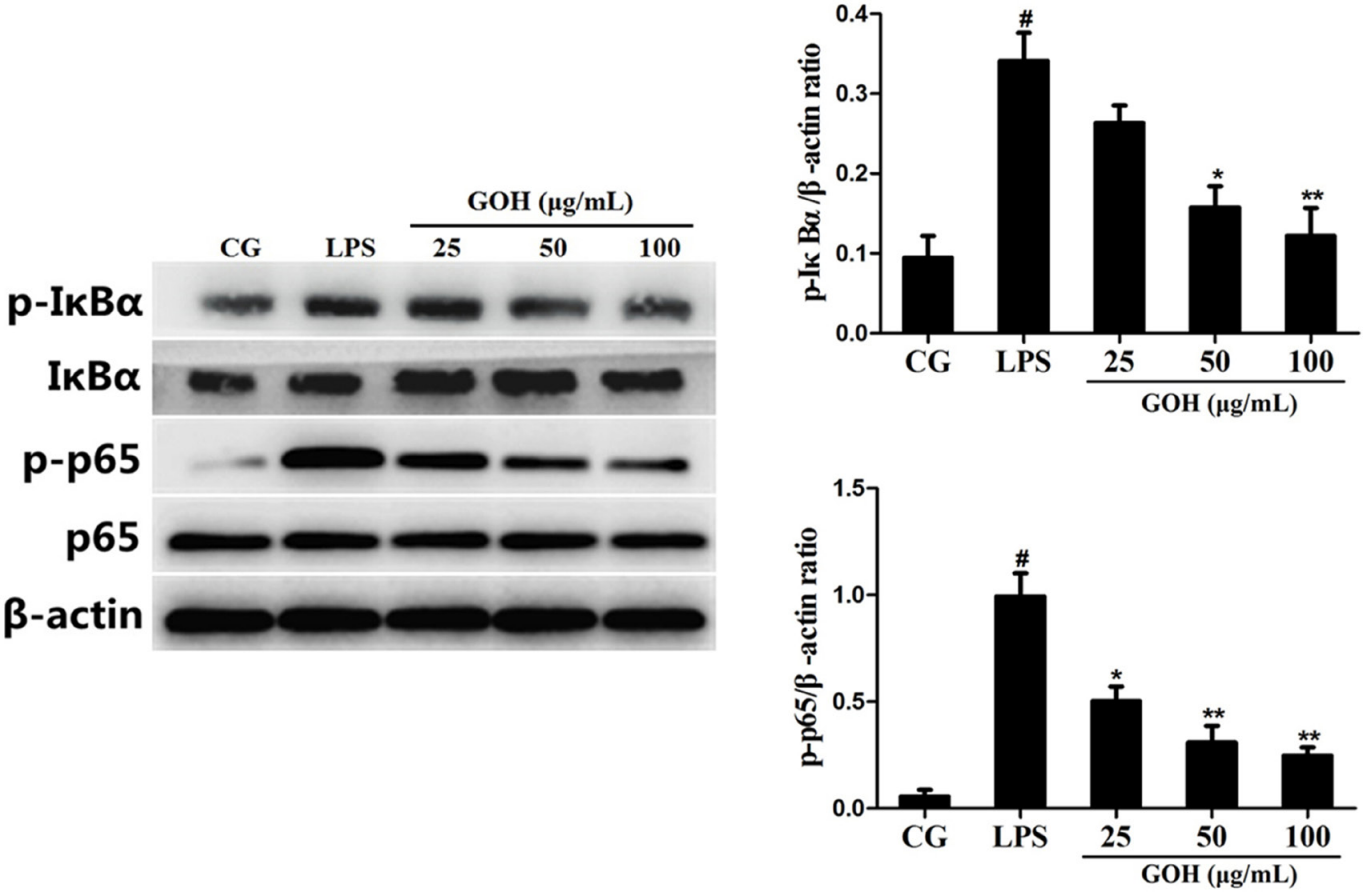
Effects of GOH on the NF-κB pathway activation in LPS-stimulated RAW 264.7 cells The levels of IκBα and p65 proteins were measured by western blotting. β-actin was used as a control. CG is the control group. LPS is the LPS-stimulated group. The values are presented as means ± S.E.M. of three independent experiments. #p<0.05 vs. the control group. *p<0.05 vs. the LPS group, **p<0.01 vs. the LPS group.

**Figure 10:**
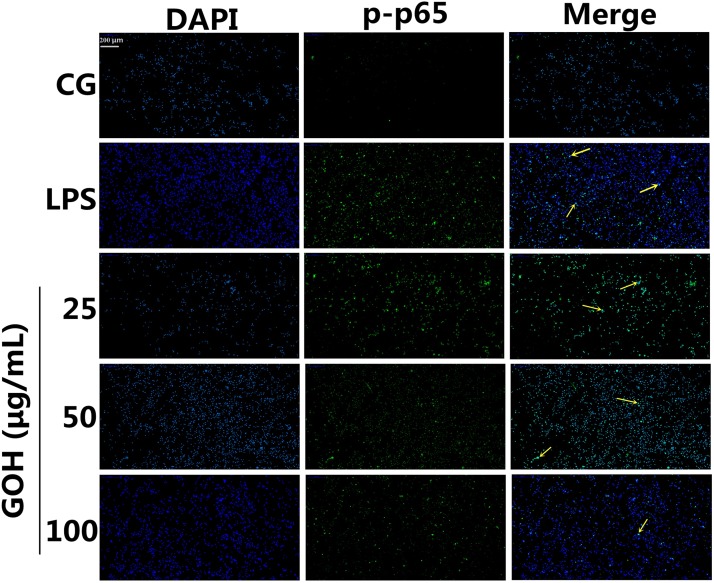
Effects of GOH on NF-κB p65 translocation into the nucleus RAW 264.7 cells were pretreated with GOH (25, 50, or 100 μg/mL) for 1 h before stimulation with LPS (1 μg/mL). The nuclear translocation of NF-κB p65 was examined with a rabbit anti-NF-κB p65 antibody and an FITC-labelled goat anti-rabbit IgG antibody, and the cells were observed with a fluorescence microscope. Scale bar: 200 μm. The yellow arrows indicate the nuclear translocation of p65. CG is the control group. LPS is the LPS-stimulated group. The values are presented as means ± S.E.M. of three independent experiments.

NF-κB is a pivotal nuclear transcription factor in the inflammatory process and can be activated by TLR4 [23, 24]. To further explore the anti-inflammatory mechanisms of GOH, we assessed the activation of the NF-κB pathway in LPS-stimulated RAW 264.7 cells. In the LPS group, LPS significantly induced phosphorylation of the p65 and IκBα proteins. However, the levels of phosphorylated p65 and IκBα were markedly reduced by GOH treatment (Figure 9). Moreover, further experiments were performed to evaluate the nuclear translocation of NF-κB p65 using immunofluorescence staining. As shown in Figure 10, LPS exposure led to the translocation of the NF-κB p65 from the cytosol to the nucleus. However, GOH treatment effectively suppressed NF-κB p65 nuclear translocation.

#### GOH treatment prevents LPS-induced apoptosis in RAW 264.7 cells

**Figure 11:**
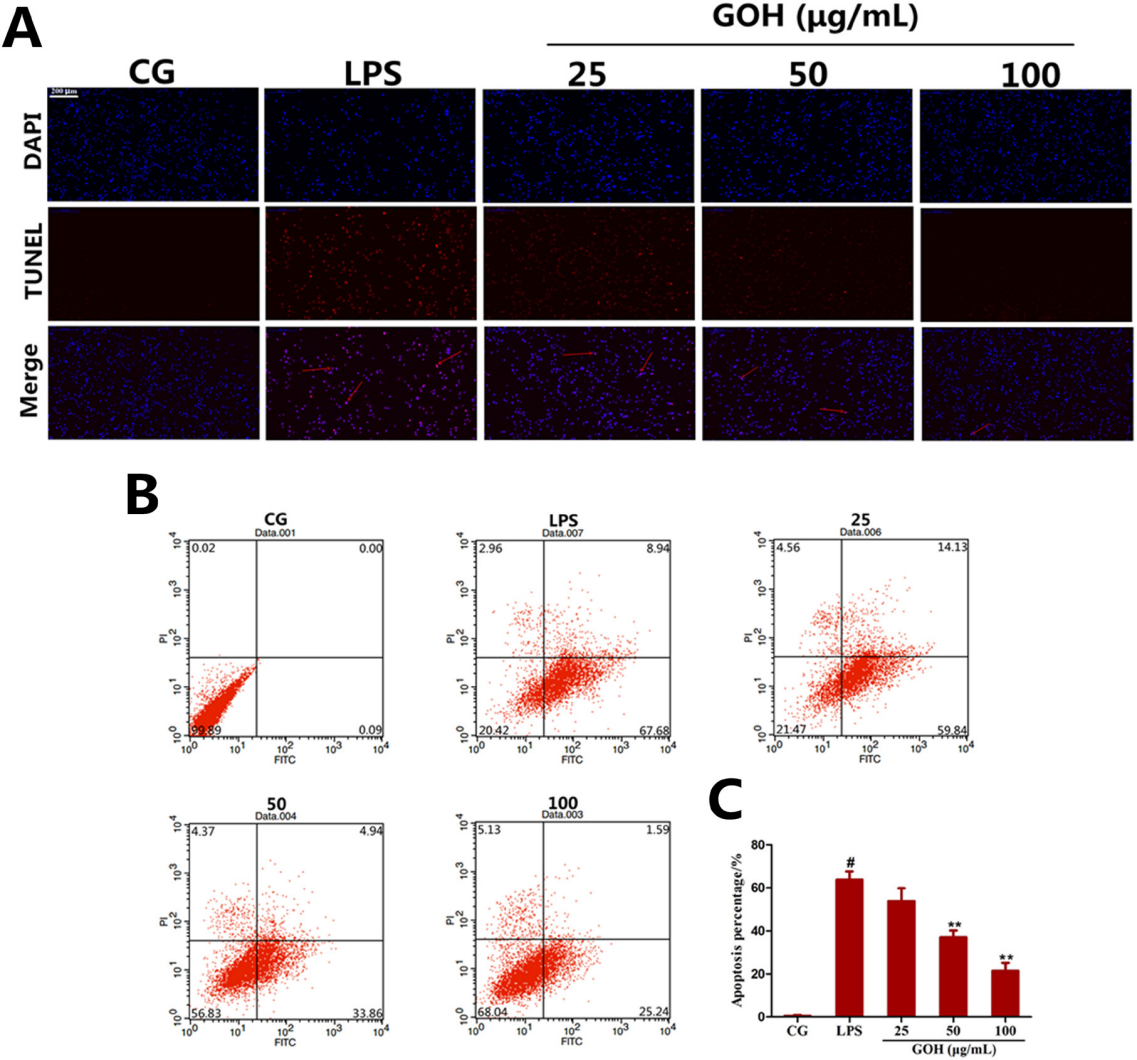
Effects of GOH on cell apoptosis induced by LPS in RAW 264. 7 cells **(A)** Inhibition of LPS-induced cell apoptosis by GOH treatment was detected by the TUNEL assay. Scale bar: 200 μm. Blue cells were nonapoptotic cells, and blue cells with red nuclei were apoptotic cells. **(B)** The cells were stained with Annexin V-FITC and PI and the apoptosis rate of cells was analysed by flow cytometry. *Lower left quadrant*, viable cells (Annexin-negative and PI-negative); *lower right quadrant*, early apoptotic cells (Annexin-positive and PI-negative); *upper right quadrant*, dead cells (Annexin-positive and PI-positive). **(C)** Apoptosis percentage. CG is the control group. LPS is the LPS-stimulated group. The values are presented as means ± S.E.M. of three independent experiments. #p<0.05 vs. the control group. *p<0.05 vs. the LPS group, **p<0.01 vs. the LPS group.

*In vivo* experiments also implied that GOH may possess a potential anti-apoptotic effect. To confirm this hypothesis, a TUNEL assay was used to detect whether GOH exhibited an anti-apoptotic effect in LPS-stimulated RAW 264.7 cells, and the results showed that the number of TUNEL-positive cells was significantly increased by LPS stimulation and was remarkably decreased by GOH treatment (Figure 11A). The anti-apoptotic effect of GOH was further confirmed by flow cytometry (Figure 11B). Flow cytometry results indicated that LPS obviously induced apoptosis, while GOH significantly reduced the percentage of apoptotic cells (Figure 11C).

#### GOH treatment reduces the expression of apoptosis-related proteins in RAW 264.7 cells

**Figure 12:**
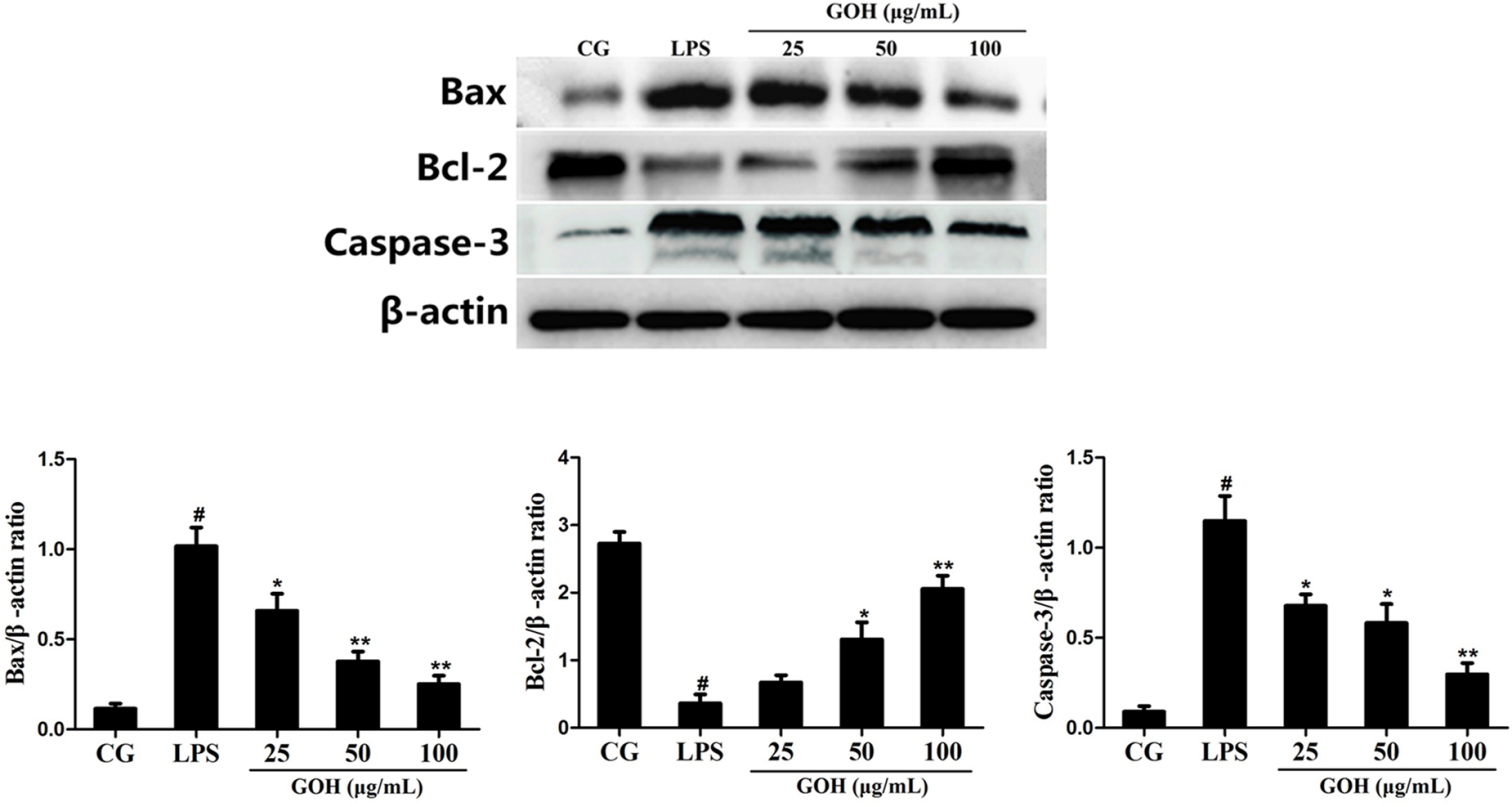
Effects of GOH on the levels of apoptosis-related proteins Bax, Bcl-2, and Caspase-3 in LPS-stimulated RAW 264.7 cells The levels of Bax, Bcl-2, and Caspase-3 proteins were measured by western blotting. β-actin was used as a control. CG is the control group. LPS is the LPS-stimulated group. The values are presented as means ± S.E.M. of three independent experiments. #p< 0.05 vs. the control group. *p< 0.05 vs. the LPS group, **p<0.01 vs. the LPS group.

Bcl-2, Bax and Caspase-3 are closely related to apoptosis [25, 26]. As shown in Figure 12, the LPS group displayed a high expression of Bax and cleaved Caspase-3 proteins compared with that in the control group. Additionally, their expression was reduced in the GOH treatment groups. Instead, the level of Bcl-2 protein was significantly downregulated in the LPS group. Additionally, its level was increased by GOH treatment in a concentration-dependent manner. These results indicated that GOH also plays an anti-apoptotic role in LPS-stimulated RAW 264.7 cells.

#### The effects of GOH are mediated through TLR4

**Figure 13:**
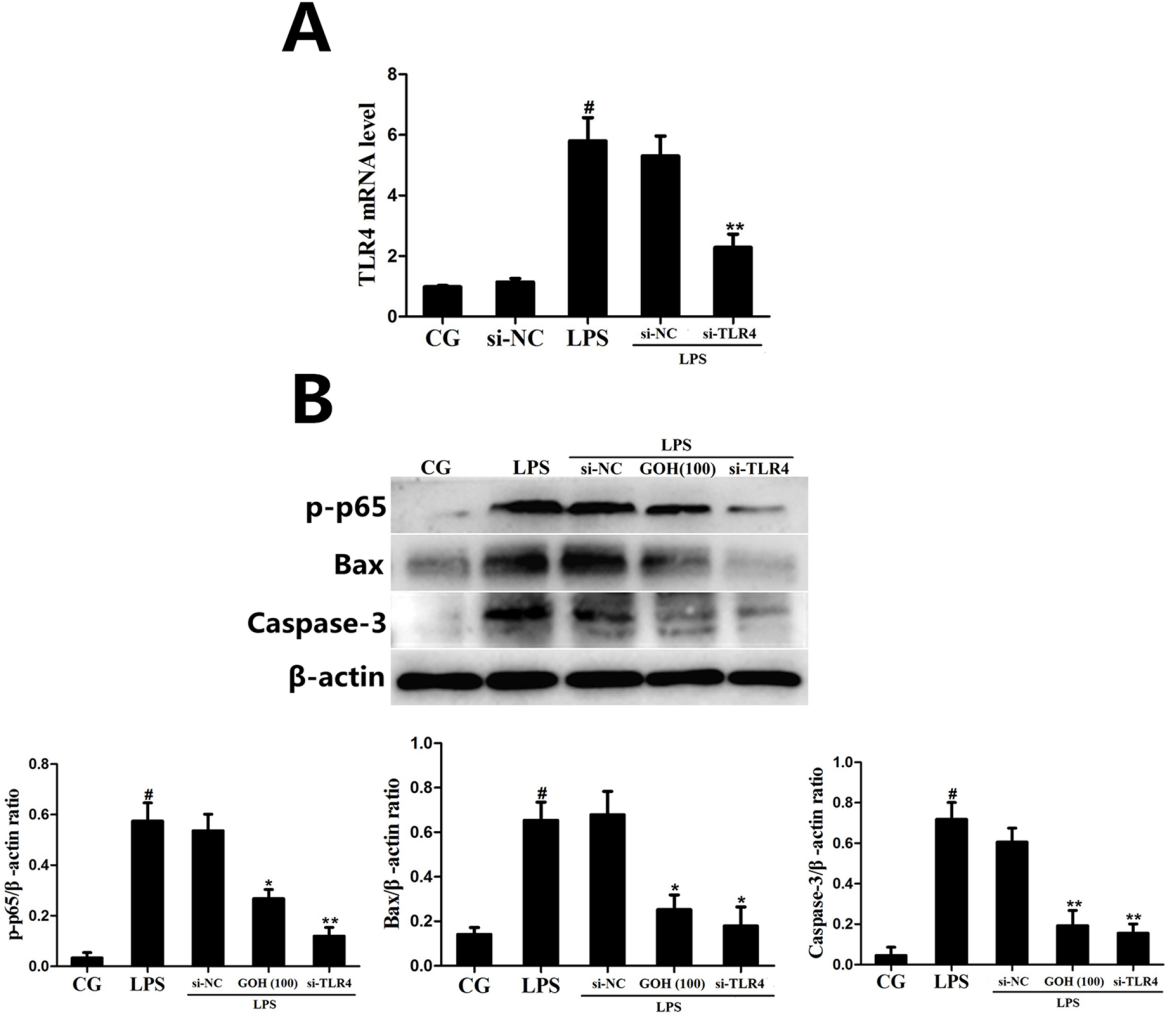
The effects of GOH are mediated through TLR4 **(A)** The interfering efficiency of TLR4 siRNA was determined using qPCR. GAPDH was used as a control. **(B)** The phosphorylation of p65, as well as the Bax and Caspase-3 levels in LPS-stimulated RAW 264.7 cells were measured by western blotting after TLR4 knockdown. β-actin was used as a control. CG is the control group. LPS is the LPS-stimulated group. The values are presented as means ± S.E.M. of three independent experiments. #p<0.05 vs. the control group. *p<0.05 vs. the LPS group, **p<0.01 vs. the LPS group.

Additionally, to further confirm whether the anti-inflammatory and anti-apoptotic effects of GOH were mediated through TLR4, a specific siRNA of TLR4 (si-TLR4) was used to knockdown TLR4 expression, and then, the phosphorylation of p65, as well as the Bax and Caspase-3 levels in RAW 264.7 cells, were measured by western blotting. As shown in Figure 13, LPS-induced phosphorylation of p65 was decreased by si-TLR4 and GOH (100 μg/mL), and the high expression of Bax and Caspase-3 induced by LPS was also subsequently reduced. The above results indicated that the anti-inflammatory and anti-apoptotic effects of GOH are mediated through TLR4.

## DISCUSSION

ALI, which is mainly characterized by pulmonary inflammation and apoptosis, is a common clinical disease in humans [8], and the mortality rate remains high at approximately 30–50% [27]. GOH, a special type of acyclic monoterpene alcohol, has been widely used to treat many diseases related to inflammation, including dextran sulfate sodium (DSS)-induced colitis in mice [28]. Moreover, GOH has been shown to inhibit cell apoptosis in traumatic spinal cord injury (SCI) [29]. In our study, a mouse model of ALI was successfully established by the intranasal administration of LPS, and we investigated the anti-inflammatory and anti-apoptotic effects of GOH on LPS-induced ALI in mice.

In the present study, the histological results showed that exposure to LPS caused serious pathological lesions, which were alleviated by GOH treatment. Additionally, we measured the lung W/D weight ratio to quantify the degree of pulmonary oedema. We observed that GOH attenuates the development of pulmonary oedema, as determined by the notable reduction in the lung W/D weight ratio. MPO is an enzyme stored in the cytoplasmic granules of neutrophils, and its activity reflects the infiltration of neutrophils into lung tissues [30]. As expected, GOH significantly decreased the activity of MPO induced by LPS, indicating that GOH could suppress neutrophil recruitment in lung tissues.

A complicated network of inflammatory cytokines, including TNF-α, IL-1β, and IL-6, plays a vital part in LPS-induced ALI and promotes the severity of lung damage [31]. Meanwhile, these cytokines also contribute to the production of various chemokines and promote the massive recruitment of neutrophils, ultimately leading to a sharp increase in MPO activity. Macrophages are an important class of immune cells, and RAW 264.7 murine macrophages have been widely employed to mimic the inflammatory response in ALI *in vitro*; thus, we explored the effects of GOH on LPS-stimulated RAW264.7 cells. In this study, we found that GOH significantly downregulated the levels of TNF-α, IL-1β, and IL-6 after LPS challenge, a finding that is consistent with the MPO results. In addition, the expression of other inflammatory mediators iNOS and COX-2 was also reduced by GOH treatment. All the above results demonstrated that GOH suppresses LPS-induced inflammation through decreasing the production of inflammatory mediators.

It is well-known that the innate immune system recognizes invasive pathogens through the activation of a series of pattern recognition receptors (PRRs) [32]. TLR4, one of the important PRRs, plays a critical role in the innate immune response against bacterial infections. Once stimulated by LPS, TLR4 interacts with its adaptor proteins, ultimately resulting in the activation of NF-κB. NF-κB is an important nuclear transcription factor that regulates various genes involved in inflammatory responses and cell survival. The activation of NF-κB involves the phosphorylation of IκBs. Once IκBα is phosphorylated, free NF-κB p65 translocates to the nucleus, and then induces the transcription of pro-inflammatory cytokines [33]. The present results show that GOH decreases the expression of TLR4, as well as the phosphorylation of NF-κB p65 and IκBα in LPS-stimulated RAW 264.7 cells.

It has been well established that an exaggerated inflammatory response is widely demonstrated to cause apoptosis, which plays a critical role in the pathological process of inflammatory diseases [34]. The infiltration and accumulation of inflammatory cells in the alveolar cavity has been reported to contribute to apoptosis and necrosis of pulmonary cells [35, 36]. Additionally, cell apoptosis was also regarded as an important component of lung injury, and the inhibition of apoptosis was demonstrated to potentially reduce ARDS morbidity [37]. In the present study, we first evaluated the anti-apoptotic effects of GOH *in vivo* using the TUNEL assay, and the results indicated that GOH treatment alleviates lung cell apoptosis *in vivo*. Subsequently, we also performed the TUNEL assay in LPS-stimulated RAW 264.7 cells. The results displayed a substantial reduction of apoptotic cells after GOH administration that was further supported by flow cytometry.

TLR4 is not only involved in an LPS-induced inflammatory response but also triggers downstream apoptotic pathways, such as Bax, Bcl-2, and Caspase-3 [25]. Bax is a pro-apoptotic member and has been reported to promote cytochrome c release and induce the activation of the downstream caspase family through the intrinsic death pathway [38]. By contrast, Bcl-2 is the best characterized anti-apoptotic protein and can inhibit the release of cytochrome c and cell death [39]. In our study, GOH decreased the protein level of Bax and increased the protein level of Bcl-2, resulting in a reduction in the ratio of Bax to Bcl-2, and ultimately attenuating aopotosis. Caspase-3 normally exists in the cytoplasm in an inactive form. When cells undergo apoptosis, Caspase-3 is activated proteolytically into cleaved Caspase-3, a biomarker of apoptotic activity. Our results also found that GOH significantly blocked the cleavage of Caspase-3 in LPS-stimulated RAW 264.7 cells. Furthermore, we confirmed the role of TLR4 using si-TLR4; the results were identical to GOH treatment (100 μg/mL) and suggested that the anti-inflammatory and anti-apoptotic effects of GOH are mediated through TLR4.

In summary, GOH treatment alleviated LPS-induced acute lung injury by suppressing pulmonary inflammation and apoptosis. The possible mechanisms for the protective role of GOH in LPS-induced ALI are associated with the inhibition of TLR4-mediated NF-κB and Bcl-2/Bax signalling pathways.

## MATERIALS AND METHODS

### Animals and reagents

Male BALB/c mice weighing 25 to 30 g were purchased from the Wuhan Institute of Biological Products Co Ltd. (Wuhan, China). All animals were housed in a specific pathogen-free room at 25 °C under a 12-h light-dark cycle and received food and water *ad libitum*. All experimental procedures were performed in accordance with the US NIH guidelines for the care and use of laboratory animals and were approved by the Ethical Committee on Animal Research at Huazhong Agricultural University.

LPS (*Escherichia coli* 055:B5) was obtained from Sigma Chemical Co. (St. Louis, MO, USA). GOH was purchased from Shanghai Yuanye Bio-Technology Co., Ltd. (Shanghai, China). The myeloperoxidase (MPO) determination kits were purchased from Nanjing Jiancheng Bioengineering Institute (Nanjing, China). All the antibodies were obtained from Cell Signalling Technology (Beverly, MA, USA). Other chemicals were of reagent grade.

### High-performance liquid chromatography (HPLC)

The purity of GOH was measured by HPLC (Figure 1B). The experiment was carried out using an EChrom2000 DAD data system (Elite, Dalian, China) as described previously.

### Mouse model of LPS-induced ALI

The method for establishing the LPS-induced ALI model was performed as previously described [40, 41]. Briefly, mice were slightly anaesthetised with an intraperitoneal injection of pentobarbital sodium, and then 50 μL of LPS (1 μg/μL) was instilled intranasally to induce lung injury. Control mice were intranasally administered 50 μL of sterile phosphate-buffered saline (PBS). 24 h after inducing infection, the mice in the GOH groups were intraperitoneally injected with 12.5, 25, and 50 mg/kg GOH three times at 0, 6, 12 h. The doses of GOH used in the study were established based on previous studies [42] and our preliminary experiments. The mice were euthanized by CO_2_ inhalation at 18 h (6 h after the last treatment with GOH), and the lung tissues were harvested and stored at -80 °C for subsequent analysis.

### Histological analysis

To evaluate the histological alterations, lung tissues were fixed with 10% buffered formalin for 24 h, embedded in paraffin, and sectioned at 4-μm thickness. After deparaffinization and dehydration, the sections were stained with haematoxylin and eosin (H&E) using standard histological techniques and then were observed by light microscopy (Olympus, Japan).

### Measurement of the W/D weight ratio

To quantify the degree of pulmonary oedema, we measured the W/D weight ratio of lung tissues. The wet lung was harvested and weighed to obtain the wet weight. Next, the lung was dried at 80°C for 48 h to obtain the dry weight. The ratio of the W/D weight was calculated by dividing the wet weight by the dry weight.

### MPO activity assay

MPO activity was defined as the quantity of enzyme degrading 1 μmol of peroxide/minute at 37°C [43]. Lung tissues were homogenized with reaction buffer (w/v 1/9), and the MPO activity was measured using an MPO determination kit following the manufacturer's instructions.

### Cell culture and treatment

RAW 264.7 cells were obtained from the American Type Culture Collection (ATCC TIB-71™). The cells were cultured in DMEM/F12 supplemented with 10% FBS and were incubated at 37 °C and 5% CO_2_. The cells were pre-treated with GOH (25, 50, or 100 μg/mL) for 1 h and then were stimulated with LPS (1 μg/mL) for 6 h. Untreated cells served as a control.

### Cell viability assay

RAW 264.7 cells were plated at a density of 1 × 10^5^ cells/mL in 96-well plates at 37 °C. 1 h later, the cells were treated with GOH at the dose of 25, 50, or 100 μg/mL and then were cultured for 24 h. Subsequently, the cell viability was examined by the 3-(4,5-dimethylthiazol-2-yl)-2,5-diphenyl tetrazolium bromide (MTT) assay. Finally, the optical density (OD) was read at 570 nm using a microplate reader (Bio-Rad Instruments, Hercules, CA, USA).

### ELISA assay

The effects of GOH on the expression of LPS-induced pro-inflammatory cytokines were measured in tissues and cells. The supernatants of the cells with different treatments were harvested. The protein levels of TNF-α, IL-1β and IL-6 in the supernatants were detected using ELISA kits (Bio-Swamp, Wuhan, China) according to the manufacturer’s instructions.

### Quantitative PCR analysis

Total RNA was extracted from the tissues and cells using TRIzol (Invitrogen, USA), following the manufacturer’s directions. Subsequently, cDNA was generated using a reverse transcription kit (Takara, Japan). qPCR was performed using the SYBR green Plus reagent kit (Roche, Basel, Swiss) and 7500 Fast Real-Time PCR System (Applied Biosystems, USA). The primers used for qPCR are listed in Table 1. The expression levels of target genes were normalized to GAPDH levels using the 2^-ΔΔCt^ method.

**Table 1:** Primers Used for qPCR

name	Primer sequence (5′-3′)	GenBank accession number	Product size (bp)
TLR4	TTCAGAGCCGTTGGTGTATC	NM_021297.2	170
	CTCCCATTCCAGGTAGGTGT		
TNF-α	CTTCTCATTCCTGCTTGTG	NM_013693.3	198
	ACTTGGTGGTTTGCTACG		
IL-1β	CCTGGGCTGTCCTGATGAGAG	NM_008361.4	131
	TCCACGGGAAAGACACAGGTA		
IL-6	GGCGGATCGGATGTTGTGAT	NM_031168.1	199
	GGACCCCAGACAATCGGTTG		
GAPDH	CAATGTGTCCGTCGTGGATCT	NM_001289726.1	124
	GTCCTCAGTGTAGCCCAAGATG		

### TUNEL assay

After routine deparaffinization and dehydration, the lung tissue sections were digested with 20 μg/mL proteinase K for 15 minutes. Endogenous peroxidase activity was blocked by 3% hydrogen peroxide for 5 min. RAW 264.7 cells in 6-well plates were incubated with control media or 1 μg/mL LPS for 6 h in the absence or presence of GOH (25, 50, or 100 μg/mL). TUNEL assay was performed in tissue sections and cells using a TUNEL assay kit according to the supplied protocol. Finally, the samples were stained with DAPI for 30 min to evaluate the cell nucleus. DAPI stains both apoptotic and nonapoptotic cells, and the apoptotic cells were recognized with dual TUNEL and DAPI staining.

### Flow cytometry

The cells at a density of 5 × 10^5^ cells/well in 6-well plates were incubated with control media or 1 μg/mL LPS for 6 h in the absence or presence of GOH (25, 50, or 100 μg/mL). At the end of treatment, the cells were collected, washed three times with ice-cold PBS and subsequently treated with 100 μL of binding buffer. Next, 5 μL of Annexin V-FITC and 5 μL of PI were added and mixed, respectively; Cells were incubated in the dark at room temperature for 15 min, and then the apoptosis rate was measured using a flow cytometry (Becton–Dickinson, San Jose, CA, USA).

### Transfection with siRNA

siRNA against TLR4 (si-TLR4) and its negative control (si-NC) were designed and synthesized (Shanghai R&S Biotechnology Co., Ltd., Shanghai, China). RAW 264.7 cells were plated at a density of 5 × 10^5^ cells/well in 6-well plates approximately 24 h prior to transfection. The cells were transfected with 200 nM si-TLR4 or si-NC using Lipofectamine 2000 (Invitrogen, Carlsbad, CA, USA), according to the manufacturer's instructions. After 6 h, the cells were treated with GOH (100 μg/mL) for 1 h, followed by LPS stimulation. After 6 h of incubation, cells were prepared for qPCR and western blotting.

### Western blot analysis

The total protein of the cells was extracted with RIPA reagent (Biosharp, China) according to the manufacturer’s recommended protocol. The protein concentration was determined by the BCA protein assay kit (Thermo Scientific, MA, USA). For western blot analysis, samples with equal amounts of protein (25 μg) were separated on 10% SDS polyacrylamide gels, were electrotransferred to polyvinylidene difluoride (PVDF) membranes, and then were blotted with primary antibody at 4 °C overnight. Next, the membranes were incubated with secondary antibody for 2 h at room temperature, and the protein expression was detected using an enhanced chemiluminescence reagent. β-actin was used as a loading control.

### Immunofluorescence staining

The nuclear translocation of NF-κB p65 in RAW 264.7 cells was detected by immunofluorescence staining. RAW 264.7 cells were maintained on 12-mm polylysine-coated glass slides for 24 h and then were stimulated with 1 μg/mL LPS with or without GOH (25, 50, or 100 μg/mL) added before stimulation. The cells were fixed with 4% paraformaldehyde for 15 min, permeabilized with 0.2% Triton X-100 for 10 min and then blocked with 5% BSA for 1 h, followed by incubation with rabbit anti-NF-κB p65 antibody overnight at 4 °C. Cells were then washed and incubated with the FITC-labelled goat anti-rabbit IgG antibody for 1 h. Nuclei were stained with DAPI for 10 min, and the NF-κB p65 subunit was observed using a fluorescence microscope (Olympus, Japan).

### Statistical analysis

All values are presented as the means ± S.E.M. The intergroup differences were analysed using one-way ANOVA followed by the LSD-*t* test for multiple comparisons. A value of p ≤ 0.05 was considered statistically significant.

## References

[1] Mendez JL, Hubmayr RD (2005). New insights into the pathology of acute respiratory failure. Curr Opin Crit Care.

[2] Artigas A, Bernard GR, Carlet J, Dreyfuss D, Gattinoni L, Hudson L, Lamy M, Marini JJ, Matthay MA, Pinsky MR, Spragg R, Suter PM (1998). The American-European Consensus Conference on ARDS, part 2: Ventilatory, pharmacologic, supportive therapy, study design strategies, and issues related to recovery and remodeling. Acute respiratory distress syndrome. Am J Respir Crit Care Med.

[3] Zhang LP, Zhao Y, Liu GJ, Yang DG, Dong YH, Zhou LH (2017). Glabridin attenuates lipopolysaccharide-induced acute lung injury by inhibiting p38MAPK/ERK signaling pathway. Oncotarget.

[4] Ware LB, Matthay MA (2000). The acute respiratory distress syndrome. N Engl J Med.

[5] Bosmann M, Ward PA (2012). Role of C3, C5 and anaphylatoxin receptors in acute lung injury and in sepsis. Adv Exp Med Biol.

[6] Matthay MA, Ware LB, Zimmerman GA (2012). The acute respiratory distress syndrome. J Clin Invest.

[7] Chopra M, Reuben JS, Sharma AC (2009). Acute lung injury:apoptosis and signaling mechanisms. Exp Biol Med (Maywood).

[8] Xie K, Yu Y, Huang Y, Zheng L, Li J, Chen H, Han H, Hou L, Gong G, Wang G (2012). Molecular hydrogen ameliorates lipopolysaccharide-induced acute lung injury in mice through reducing inflammation and apoptosis. Shock.

[9] Kitamura Y, Hashimoto S, Mizuta N, Kobayashi A, Kooguchi K, Fujiwara I, Nakajima H (2001). Fas/FasL-dependent apoptosis of alveolar cells after lipopolysaccharide-induced lung injury in mice. Am J Respir Crit Care Med.

[10] Rojas M, Woods CR, Mora AL, Xu J, Brigham KL (2005). Endotoxin-induced lung injury in mice: structural, functional, and biochemical responses. Am J Physiol Lung Cell Mol Physiol.

[11] Gharib SA, Liles WC, Matute-Bello G, Glenny RW, Martin TR, Altemeier WA (2006). Computational identification of key biological modules and transcription factors in acute lung injury. Am J Respir Crit Care Med.

[12] Bosmann M, Grailer JJ, Russkamp NF, Ruemmler R, Zetoune FS, Sarma JV, Ward PA (2013). CD11c+ alveolar macrophages are a source of IL-23 during lipopolysaccharide-induced acute lung injury. Shock.

[13] Park JR, Lee H, Kim SI, Yang SR (2016). The tri-peptide GHK-Cu complex ameliorates lipopolysaccharide-induced acute lung injury in mice. Oncotarget.

[14] Xaus J, Comalada M, Valledor AF, Lloberas J, Lopez-Soriano F, Argiles JM, Bogdan C, Celada A (2000). LPS induces apoptosis in macrophages mostly through the autocrine production of TNF-alpha. Blood.

[15] Hull C, McLean G, Wong F, Duriez PJ, Karsan A (2002). Lipopolysaccharide signals an endothelial apoptosis pathway through TNF receptor-associated factor 6-mediated activation of c-Jun NH2-terminal kinase. J Immunol.

[16] Yu LC, Flynn AN, Turner JR, Buret AG (2005). SGLT-1-mediated glucose uptake protects intestinal epithelial cells against LPS-induced apoptosis and barrier defects: a novel cellular rescue mechanism?. FASEB J.

[17] Kaiser WJ, Offermann MK (2005). Apoptosis induced by the toll-like receptor adaptor TRIF is dependent on its receptor interacting protein homotypic interaction motif. J Immunol.

[18] Vinothkumar V, Manoharan S, Sindhu G, Nirmal MR, Vetrichelvi V (2012). Geraniol modulates cell proliferation, apoptosis, inflammation, and angiogenesis during 7,12-dimethylbenz[a]anthracene-induced hamster buccal pouch carcinogenesis. Mol Cell Biochem.

[19] de Cassia da Silveira e Sa R, Andrade LN, de Sousa DP (2013). A review on anti-inflammatory activity of monoterpenes. Molecules.

[20] Chaudhary SC, Siddiqui MS, Athar M, Alam MS (2013). Geraniol inhibits murine skin tumorigenesis by modulating COX-2 expression, Ras-ERK1/2 signaling pathway and apoptosis. J Appl Toxicol.

[21] Carnesecchi S, Schneider Y, Ceraline J, Duranton B, Gosse F, Seiler N, Raul F (2001). Geraniol, a component of plant essential oils, inhibits growth and polyamine biosynthesis in human colon cancer cells. J Pharmacol Exp Ther.

[22] Cheng A, Han C, Fang X, Sun J, Chen X, Wan F (2016). Extractable and non-extractable polyphenols from blueberries modulate LPS-induced expression of iNOS and COX-2 in RAW264.7 macrophages via the NF-kappaB signalling pathway. J Sci Food Agric.

[23] Wu H, Jiang K, Yin N, Ma X, Zhao G, Qiu C, Deng G (2017). Thymol mitigates lipopolysaccharide-induced endometritis by regulating the TLR4- and ROS-mediated NF-kappaB signaling pathways. Oncotarget.

[24] Kim TW, Lee SJ, Oh BM, Lee H, Uhm TG, Min JK, Park YJ, Yoon SR, Kim BY, Kim JW, Choe YK, Lee HG (2016). Epigenetic modification of TLR4 promotes activation of NF-kappaB by regulating methyl-CpG-binding domain protein 2 and Sp1 in gastric cancer. Oncotarget.

[25] Song X, Guo M, Wang T, Wang W, Cao Y, Zhang N (2014). Geniposide inhibited lipopolysaccharide-induced apoptosis by modulating TLR4 and apoptosis-related factors in mouse mammary glands. Life Sci.

[26] He Y, Mo Q, Luo B, Qiao Y, Xu R, Zuo Z, Deng J, Nong X, Peng G, He W, Wei Y, Hu Y (2016). Induction of apoptosis and autophagy via mitochondria- and PI3K/Akt/mTOR-mediated pathways by E. adenophorum in hepatocytes of saanen goat. Oncotarget.

[27] Matthay MA, Zimmerman GA, Esmon C, Bhattacharya J, Coller B, Doerschuk CM, Floros J, Gimbrone MA, Hoffman E, Hubmayr RD, Leppert M, Matalon S, Munford R (2003). Future research directions in acute lung injury: summary of a National Heart, Lung, and Blood Institute working group. Am J Respir Crit Care Med.

[28] De Fazio L, Spisni E, Cavazza E, Strillacci A, Candela M, Centanni M, Ricci C, Rizzello F, Campieri M, Valerii MC (2016). Dietary Geraniol by Oral or Enema Administration Strongly Reduces Dysbiosis and Systemic Inflammation in Dextran Sulfate Sodium-Treated Mice. Front Pharmacol.

[29] Wang J, Su B, Zhu H, Chen C, Zhao G (2016). Protective effect of geraniol inhibits inflammatory response, oxidative stress and apoptosis in traumatic injury of the spinal cord through modulation of NF-kappaB and p38 MAPK. Exp Ther Med.

[30] Klebanoff SJ (2005). Myeloperoxidase: friend and foe. J Leukoc Biol.

[31] Goodman RB, Pugin J, Lee JS, Matthay MA (2003). Cytokine-mediated inflammation in acute lung injury. Cytokine Growth Factor Rev.

[32] Akira S, Takeda K, Kaisho T (2001). Toll-like receptors: critical proteins linking innate and acquired immunity. Nat Immunol.

[33] Jiang K, Chen X, Zhao G, Wu H, Mi J, Qiu C, Peng X, Deng G (2017). IFN-tau Plays an Anti-Inflammatory Role in Staphylococcus aureus-Induced Endometritis in Mice Through the Suppression of NF-kappaB Pathway and MMP9 Expression. J Interferon Cytokine Res.

[34] Oyinloye BE, Adenowo AF, Kappo AP (2015). Reactive oxygen species, apoptosis, antimicrobial peptides and human inflammatory diseases. Pharmaceuticals.

[35] Jing W, Chunhua M, Shumin W (2015). Effects of acteoside on lipopolysaccharide-induced inflammation in acute lung injury via regulation of NF-kappaB pathway *in vivo* and *in vitro*. Toxicol Appl Pharmacol.

[36] Li Y, Xiao J, Tan Y, Wang J, Zhang Y, Deng X, Luo Y (2017). Inhibition of PKR ameliorates lipopolysaccharide-induced acute lung injury by suppressing NF-kappaB pathway in mice. Immunopharmacol Immunotoxicol.

[37] Galani V, Tatsaki E, Bai M, Kitsoulis P, Lekka M, Nakos G, Kanavaros P (2010). The role of apoptosis in the pathophysiology of Acute Respiratory Distress Syndrome (ARDS): an up-to-date cell-specific review. Pathol Res Pract.

[38] Rosse T, Olivier R, Monney L, Rager M, Conus S, Fellay I, Jansen B, Borner C (1998). Bcl-2 prolongs cell survival after Bax-induced release of cytochrome c. Nature.

[39] Tsujimoto Y (1998). Role of Bcl – 2 family proteins in apoptosis: apoptosomes or mitochondria?. Genes Cells.

[40] Zhang X, Song K, Xiong H, Li H, Chu X, Deng X (2009). Protective effect of florfenicol on acute lung injury induced by lipopolysaccharide in mice. Int Immunopharmacol.

[41] Wu H, Zhao G, Jiang K, Chen X, Rui G, Qiu C, Guo M, Deng G (2016). IFN-tau Alleviates Lipopolysaccharide-Induced Inflammation by Suppressing NF-kappaB and MAPKs Pathway Activation in Mice. Inflammation.

[42] La Rocca V, da Fonseca DV, Silva-Alves KS, Ferreira-da-Silva FW, de Sousa DP, Santos PL, Quintans-Junior LJ, Leal-Cardoso JH, de Almeida RN (2017). Geraniol Induces Antinociceptive Effect in Mice Evaluated in Behavioural and Electrophysiological Models. Basic Clin Pharmacol Toxicol.

[43] Cuzzocrea S, McDonald MC, Mazzon E, Dugo L, Serraino I, Threadgill M, Caputi AP, Thiemermann C (2002). Effects of 5-aminoisoquinolinone, a water-soluble, potent inhibitor of the activity of poly (ADP-ribose) polymerase, in a rodent model of lung injury. Biochem Pharmacol.

